# Correlation in chicken between the marker LEI0258 alleles and Major Histocompatibility Complex sequences

**DOI:** 10.1186/1753-6561-5-S4-S29

**Published:** 2011-06-03

**Authors:** Olympe Chazara, Helle Risdahl Juul-Madsen, Chi-Sheng Chang, Michele Tixier-Boichard, Bertrand Bed’hom

**Affiliations:** 1 UMR 1313 Génétique Animale et Biologie Intégrative, INRA/AgroParisTech, 78352 Jouy en Josas, France; 2 Department of Animal Health and Bioscience, Faculty of Agricultural Sciences, University of Aarhus, DK-8830 Tjele, Denmark; 3 Department of Animal Science, National Chung-Hsing University, Taiwan, R.O.C

## Abstract

**Background:**

The LEI0258 marker is located within the B region of the chicken Major Histocompatibility Complex (MHC), and is surprisingly well associated with serology. Therefore, the correlation between the LEI0258 alleles and the MHC class I and the class II alleles at the level of sequences is worth investigating in chickens. Here we describe to which extent the LEI0258 alleles are associated with alleles of classical class I genes and non-classical class II genes, in reference animals as well as local breeds with unknown MHC haplotypes.

**Methods:**

For the class I region, in an exploratory project, we studied 10 animals from 3 breeds: Rhode Island Red, White Leghorn and Fayoumi chickens, by cloning and sequencing *B-F1* and *B-F2* cDNA from exon 1 to 3’UTR. For the class II region, we reconstructed haplotypes of the 8.8 kb genomic region encompassing three non-classical class II genes: *B-DMA*, *B-DMB1* and *B-DMB2*, for 146 animals from more than 50 breeds including wild species of jungle fowls.

**Results:**

Overall we found that the LEI0258 marker genotypes gave good indications of the MHC haplotypes, and a very good predictions (>0.95) of the heterozygosity of an animal at the MHC locus.

**Conclusions:**

Our results show that the LEI0258 alleles are strongly associated with haplotypes of classical class I genes and non-classical class II genes, unravelling the reasons why this marker is becoming the reference marker for MHC genotyping in chickens.

## Background

Classical MHC class I molecules are membrane proteins expressed on all nucleated cells. They are heterodimers consisting of a highly polymorphic heavy chain α and a β2-microglobulin light-chain. Classical MHC I α chains are highly polymorphic and implicated in the binding of small peptides derived from the intracellular compartment. The peptides are bound in a groove of the molecule formed by the two polymorphic domains α1 and α2, respectively encoded by exons 2 and 3 of the class Ia gene. Classical MHC class I molecules interact with T cell receptors (TCR) of cytotoxic T lymphocytes and receptors on natural killer cells.

Classical MHC class II molecules consist of two heavy chains, α and β, primarily expressed by B cells, macrophages and activated T-cells. They present peptides, primarily derived from exogenous molecules, to T helper lymphocytes. Non-classical class II DM molecules also comprise two heavy chains, α and β. They are involved in the formation of classical class II/peptide complexes, before their expression on the surface of the cell. It has been shown that without DM, these complexes are unstable, disrupting antigen presentation [1].

**Figure 1:**
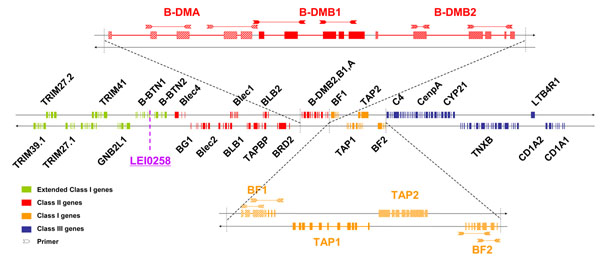
Core chicken MHC B region showing gene content and positions of the primers used for PCR

The chicken MHC B class I region comprises two genes, encoding class Iα chains: *B-F1* (*B-FI* or *B-F* minor) and *B-F2* (*B-FIV* or *B-F* major). The *B-F2* gene is described as the major expressed locus. In addition, the chicken MHC B class II region comprises three genes encoding non-classical DM chains, one α chain and two β chains: respectively *B-DMA*, *B-DMB1* and *B-DMB2* (Figure 1). At the border of the class II region, one atypical variable number of tandem repeat (VNTR) marker has been reported: the LEI0258 marker, which is highly polymorphic, with 26 allele sizes already reported [2].

The aim of this study was to evaluate the feasibility of full cloning and sequencing of the two classical class I *B-F* genes in individuals from three different chicken breeds and to exploit previously obtained sequences of the three non-classical *B-DM* genes from more than 50 breeds in order to validate the LEI0258 marker as a predictor of MHC genotypes.

## Methods

### LEI0258 genotyping

For the classical class I *B-F* genes study, ten animals have been used: four Rhode Island Red (RIR) and three White Leghorn (WL) from experimental lines, and three Fayoumi, an Egyptian local breed. The animals were genotyped by PCR amplification for the LEI0258 marker. Primers were LEI0258-F: CACGCAGCAGAACTTGGTAAGG forward and LEI0258-R: AGCTGTGCTCAGTCCTCAGTGC reverse. Four animals were homozygous 357/357, two 381/381, one 309/309, one 307/307 and two heterozygous, one 307/309 and one 261/357.

For the non-classical class II *B-DM* genes study, 129 animals were used from 48 different populations or lines of domestic chickens, and three wild *Gallus sp*. All animals were genotyped by PCR amplification for the LEI0258 marker. Forty alleles were found, defined by their size, ranging from 182 bp to 539 bp, including the alleles 261, 307, 309, 357, and 381.

### Classical class I B-F genotyping

Caecal tonsils were conserved in RNA later (Qiagen) at –20°C prior to extraction. Total RNA was isolated from approximately half a caecal tonsil using the Rneasy Mini Kit (Qiagen) according to the manufacturer’s instructions. The reverse transcription and first strand cDNA synthesis were carried out in 33 µL using the Amersham Pharmacia Biotech kit from 2.5 µg RNA with DTT solution and pd(N)_6_ at 0.2 µg/µL as primers. PCRs were carried out from 2 µL of cDNA in a final volume of 25 µL containing 2.5 µL 10X buffer, 2 µL dNTP (2 mM each), 1.25 µL MgCl_2_ (25 mM), 1 µL of each primer (at 10 µM), and 0.5 U DNA polymerase. The PCR cycle setup was 40 cycles of 94°C for 1 min, 95°C for 1 min, 55°C for 1 min, 72°C for 2 min, followed by 72°C for 10 min. Two PCR primers pairs were used. They were designed to amplify the two *B-F* genes simultaneously. The first one amplified the first part of the *B-F* molecules (564 bp): BFα-1-8 F GAGCTCCATACCCTGCGGTACATC and BFα-180-189 R CCCCCACACTCGCACCTCGGGCCGCTC (PCR1), and the second one amplified the last part of the *B-F* molecules (850 bp): BF-exon-3A F TTCCAGAGGCAGTTCCCACCAA and BF-EXON-8b R AGCCAAACTGGGACACGGTC (PCR2) (Figure 1).

Two µL of the purified PCR products were cloned into the pCR 2.1-TOPO vector using the TOPO TA Cloning Kit and One Shot Mach1™-T1R chemically competent *E. coli* (Invitrogen) according to the manufacturer’s instructions. Positive clones were tested by PCR amplification using custom ordered primers : TOPO M13 forward GTAAAACGACGGCCAG and TOPO M13 reverse CAGGAAACAGCTATGAC, in 25 µL final volume, from 1 µL liquid culture, with 2.5 µL 10X buffer, 2.5 µL dNTP (2 mM each), 2.5 µL of each primer (at 3 µM), and 0.5 U DNA polymerase. The PCR cycle setup was 95°C for 3 min followed by 40 cycles of 95°C for 45 s, 55°C for 1 min, 72°C for 1 min, followed by 72°C for 10 min. When needed, the resulting PCRs were purified with the QIAquick PCR Purification Kit (Qiagen), before sequencing was carried out in both directions by Eurofins MWG Operon, using their standard protocol for purified PCR products, with the previously described TOPO M13 primers. Analysis of sequence quality and variation analyses were carried out with novoSNP [3], and Pregap4 v1.5 and Gap4 v4.10 components of the Staden package [4].

### Non-classical class II B-DM genotyping

Six primers have been used to resequence the 3 *B-DM* genes from genomic DNA [5], including exon 2 and exon 3 (Figure 1). Individual genotypes were obtained for the 129 individuals and completed using the 16 corresponding sequences available in GenBank (accession numbers AL023516 from [6], AB268588 from [7] and AB426141 to AB426154 from [8].

### Statistical analysis

First, heterozygosity at the LEI0258 marker level was compared with heterozygosity at the classical class I or non-classical class II sequences. Then association between LEI0258 and MHC were studied. The Spearman rank-order correlation coefficient has been calculated and tested for the non-classical class II sequences. No statistical test were used to test correlation with classical class I sequences due to the small number of animals investigated.

## Results and discussion

### Classical class I B-F gene sequences

Purified PCR fragments were obtained for the ten animals but further analysis were only obtained from seven animals. 57 sequences corresponding to a classical class Ia gene of the B region of the MHC of the chicken were obtained for PCR1 and 21 for PCR2. The primer design strategy was to have a 164 bp overlap between the two PCRs containing unambiguous polymorphisms between known sequences from the common serotypes. Thus, we were able to merge PCR1 and PCR2 sequences from each haplotype.

It is worth mentioning that for PCR2, three sequences of the class Iα gene of the Y region of the MHC of the chicken were obtained, one for animal 4472 and two for animal 4867, corresponding to the predicted gene XM_425314 of the chromosome 16 reference assembly (NC_006103).

### Nucleotide sequence variability

Regarding the classical class I *B-F* genes, we identified 141 polymorphic positions corresponding to 123 biallelic SNPs, 17 triallelic SNPs, and one 1 bp indel. Those polymorphisms defined a minimum number of eight alleles. We also observed two important indels, 33 bp and 63 bp respectively, in animal 4203. Because those deletions affected the same allele of the same animal, they are probably the consequences of splicing variations. The 33 bp deletion affecting the entire exon 7 has been observed before [9], the 63 bp deletion starting at the 3’ end of exon 3 until the 5’ end of exon 4 was never reported and included the site matching the reverse primer of PCR1, BFα-180-189 R.

Concerning the non-classical class II *B-DM* genes, we considered the 124 haplotypes previously described for the whole 8.8 kb *B-DM* region [5] reconstructed from the genotypes of 146 individuals (129 domestic and wild animals plus 16 sequences available in GenBank and the genome sequence) at 158 SNPs.

### Classical class I *B-F* genes

In order to compare the obtained sequences for the MHC classical class I *B-F* genes with sequences of standard chicken serotypes, we used the genomic sequences published by [8], corresponding to ten White-Leghorn serotypes: B2, B5, B6, B9, B12, B13, B15, B17, B19, and B21, two Ancona breed serotypes: B8 and B11, and two New Hampshire breed serotypes B23 and B24. The sequences were defined for the *B-F1* or *B-F2* gene according to their annotation in those sequences (Table 1).

The most frequent sequence, found in 34 clones from four animals, corresponded to the BF2 gene of the B21 serotype (B21-BF2). Those four animals were homozygous 357/357 with the LEI0258 marker (Fayoumi-1, WL-1, and WL-2) or homozygous 381/381 (Fayoumi-2). The 357 bp LEI0258 allele corresponds to the RIR B21 serotype. Another sequence, observed in 10 clones, matched the BF2 gene of the B24 serotype (B24-BF2) and was found in a RIR having a 309/309 genotype (RIR-2). The 309 bp LEI0258 allele corresponds, among others, to the RIR B24 serotype.

For five clones, we observed recombinant sequences corresponding to both genes. Such recombinant sequences have been reported previously in MHC genes families, where an amplified gene fragment can act as a PCR primer for another gene, leading to artefactual sequences [10,11].

Overall, those results showed that the *B-F* sequences found corresponded to sequences reported for serotypes with matching LEI0258 genotypes, even in non-WL animals (Table 1). This had been reported previously for local Brazilian Caipira chickens [12]. We also found, that the majority of the clones obtained contained the gene described as the major gene (91% for B24, 92% for B21) except for the 307/307 RIR chicken (4123) where we mostly found the allele described as the *B-F1* allele for B23. One hypothesis could be that the *B-F2* gene corresponding to this 307/307 chicken is very similar to the B23-BF1 gene.

**Table 1:** The *B-F* genes identified from the seven chickens studied

Animal	LEI0258	***B-F***	Best match	No
RIR-1	307/307	gene 1	B23-BF1	8
RIR-1	307/307	gene 2	no match	3
RIR-1	307/307	recomb	B23-BF1 & no match	1
RIR-2	309/309	gene 1	B24-BF2	8
RIR-2	309/309	gene 1	B24-BF2 + del 33 bp	1
RIR-2	309/309	gene 1	B24-BF2 + del 63bp & 33 bp	1
RIR-2	309/309	gene 2	B13, B15, B21, B24-BF1	1
Fayoumi-1	357/357	gene 1	B21-BF2	7
WL-1	357/357	gene 1	B21-BF2	8
WL-1	357/357	gene 2	B21, B15-BF1 (partial)	1
WL-2	357/357	gene 1	B21-BF2	9
WL-2	357/357	recomb	B21-BF1 & BF2	1
Fayoumi-2	381/381	gene 1	B21-BF2	10
Fayoumi-2	381/381	gene 2	B21-B15-BF1	2
Fayoumi-2	381/381	recomb	B21-BF2 & BF1	2
WL-3	261/357	gene 1	B15-BF2	13
WL-3	261/357	gene 2	no match	1

RIR, Rhode Island Red; WL, White Leghorn; LEI0258 genotypes are given in base pairs; recomb, recombinant sequence; No, number of clones sequenced.

### Non-classical class II *B-DM* genes

Correlation between the 124 *B-DM* haplotypes and the 40 LEI0258 alleles was investigated. The common *B-DM* haplotypes encountered in the study were haplotypes number 1, 34, 52, and 58, respectively corresponding primarily to LEI0258 alleles 357, 309, 261, 487 or 539. The correlation was tested for all haplotypes and was significant (p < 0.005). Finally, we were also able to compare heterozygosity at the *B-DM* locus with heterozygosity at the LEI0258 marker locus. Only six animals out of 147 were homozygotes for the LEI0258 marker but heterozygotes for the *B-DM* haplotypes. There were also two animals that were homozygotes for *B-DM* haplotypes but heterozygotes for the LEI0258 marker, so in total, the heterozygosity matched between the *B-DM* region and LEI0258 was 139 of the 147 cases (95 %).

## Conclusions

The results show that the LEI0258 can definitely help predicting MHC heterozygosity at the class I and class II locus. Furthermore, our observations reveal that even at the level of the highly polymorphic MHC genes, the LEI0258 marker is a good indicator of the MHC haplotype, probably thanks to its very high number of alleles. This means that, the LEI0258 marker can be used as a preliminary screening in any population or breed of chickens, but also in well characterized populations. In fact, this marker is increasingly used in research and breeding for planning crosses and will probably become a successful tool for controlling the animals’ MHC genotypes, an important genetic factor to take into account in breeding.

## Competing interests

The authors declare that they have no competing interests.

## Authors' contributions

OC carried out the molecular genetic studies, performed the statistical analysis and drafted the manuscript. HJM participated in the design of the study, the analysis and the interpretation of the data. CSC carried some of the molecular genetic studies and their analysis. MTB participated in the design of the study, the analysis and the interpretation of the data. BB coordinated the study, participated in its design, the analysis and the interpretation of the data. All authors contributed to the manuscript and all authors read and approved the final manuscript.
